# Peer review of "Geochemical and Statistical Analyses of Trace Elements in Lake Sediments from Qaidam Basin, Qinghai-Tibet Plateau: Distribution Characteristics and Source Apportionment"

_ijerph, 2022, doi:10.3390/ijerph19042341_

Round 1
Reviewer 1 Report
In this study, the authors measured the concentrations of trace elements in 57 sediment samples collected from six lakes in the Qaidam Basin, Qinghai-Tibet Plateau (QTP). Then, they examined the distribution characteristics and sources of trace elements in the sediments. The safety of lake ecosystems on the QTP has attracted increasing attention, owing to the unique location and ecological vulnerability. Hence, I think that the results presented here are valuable, because such data are still scarce in the QTP. However, I have doubts about the accuracy of analytical results (Table 1), as mentioned below.
Lines 104–106: The authors should provide data related to the lakes, including area, water depth, eutrophic level, and redox state in sediment.
Lines 110–111: The authors collected sediment samples using grab sampler. What is the thickness of sediments collected? Moreover, how many years does the thickness of sediments collected correspond to? It is expected that this number of years is markedly different for each lake depending on sedimentation rate. This may make it difficult to evaluate the pollution level of trace elements in each lake.
Lines 126–136: Was the accuracy of metal analysis verified using a proper standard reference material?
Table 1 and lines 202–208: The significant figures for given values are too many. They will be at most three based on analytical errors of ICP-MS and ICP-OES. Moreover, correct the background values of Cd and Ba.
Lines 227–228: Do the authors mean that Ba is saturated with a sulfate or carbonate in the lake water? The authors should give evidence to support this.
Lines 233–234: I am surprised that the unit of element concentrations in the lake sediments is μg/kg (= ppb). Is this truly correct? If so, the element concentrations presented in Table 1 are abnormally low compared with the background values (unit: mg/kg = ppm). However, this will be impossible. Thus, I have doubts about the accuracy of analytical results. Was the accuracy of metal analysis verified using a proper standard reference material?
Lines 275–277: I disagree with that As mainly derives from anthropogenic sources. The lake sediments are not polluted by As, because As concentration in the lake sediments is extremely lower than its background value.
Lines 277–278: I do not understand that in nature, Ba mainly exists as either sulfuric or carbonic acid. In addition, what is the form of adsorption or mechanical mixing? Polite explanation is needed.
Lines 339–367: The authors should give more evidence to support this source apportionment. What is the reason why the contribution of traffic emission to Cd is relatively large? Also, what is the reason why the contribution of atmospheric deposition and anthropogenic inputs to Ba is relatively large?
Author Response
Thanks for your valuable and helpful comments. We have responded comments carefully and made the necessary correction point by point. All of the revised part in our revised manuscript has been highlighted in red.
Response to Reviewer 1 Comments
Point 1: In this study, the authors measured the concentrations of trace elements in 57 sediment samples collected from six lakes in the Qaidam Basin, Qinghai-Tibet Plateau (QTP). Then, they examined the distribution characteristics and sources of trace elements in the sediments. The safety of lake ecosystems on the QTP has attracted increasing attention, owing to the unique location and ecological vulnerability. Hence, I think that the results presented here are valuable, because such data are still scarce in the QTP. However, I have doubts about the accuracy of analytical results (Table 1), as mentioned below.
Response 1: Thanks so much for your help in revision. We are grateful to you by these important points and respond to them carefully below.
Point 2: Lines 104–106: The authors should provide data related to the lakes, including area, water depth, eutrophic level, and redox state in sediment.
Response 2: Thanks for the reviewer’s kind advice. According to the reviewer’s suggestion, we have supplemented the relevant lake information such as the area and water depth of the six lakes (lines117-140). In addition, we did not test eutrophication-related indicators and redox potential when sampling in the field, so we did not conduct relevant analysis. We reviewed a lot of literature, and due to the lack of studies on lakes in the study area, we only found that the eutrophication level of Kreuk Lake was moderate (Zhou, Yang,2016). In future research, we must pay attention to this.
References:
Zhou.X.; Yang.X. Eutrophication evaluation of keluke lake based on Grey Clustering method. Journal of Qinghai Normal university. 2016,32(03): 63-70.DOI:10.16229/j.cnki.issn1001-7542.2016.03.011.
Point 3: Lines 110–111: The authors collected sediment samples using grab sampler. What is the thickness of sediments collected? Moreover, how many years does the thickness of sediments collected correspond to? It is expected that this number of years is markedly different for each lake depending on sedimentation rate. This may make it difficult to evaluate the pollution level of trace elements in each lake.
Response 3: Thanks for your valuable comments. As you pointed out, the thickness of sediments and sediment rate vary widely from lake to lake. The element contents in sediments at different depths of lake sediments contain rich geochemical information, and are often used to reflect the climatic conditions and the intensity of human activities in different years. In the present study, sediment samples were sampled from the six lakes using a grab sampler. The depth of penetration of the grab sampler was 4–10 cm into the sediment. Such sediment thicknesses have been used to study various changes in recent decades in other similar studies (Guo et al.,2015; Guo et al.,2018; Niu et al.,2020; Dai et al.,2018). This study is to reflect the impact of human activities on lakes on the Qinghai-Tibet Plateau in recent years. The depositional age of these sediments and the relevant scientific questions worth studying. We hope to do this research in future, to examine the changes in the geochemical characteristics of the Qinghai-Tibet Plateau over hundreds or even tens of thousands of years.
References:
- Guo, W.; Huo, S.; Xi, B.; Zhang, J.; Wu, F. Heavy metal contamination in sediments from typical lakes in the five geographic regions of China: Distribution, bioavailability, and risk. Ecol. Chem. Eng. 2015, 81, 243-255.
- Guo, B.; Liu, Y.; Zhang, F.; Hou, J.; Zhang, H.; Li, C. Heavy metals in the surface sediments of lakes on the Tibetan Plateau, China. Environ. Sci. Pollut. Res. 2018, 25(4), 3695-3707.
- Niu, Y.; Jiang, X.; Wang, K.; Xia, J.; Jiao, W.; Niu, Y.; Yu, H. Meta analysis of heavy metal pollution and sources in surface sediments of Lake Taihu, China. Sci. Total Environ. 2020, 700, 134509.
- Dai, L.; Wang, L.; Li, L.; Liang, T.; Zhang, Y.; Ma, C.; Xing, B. Multivariate geostatistical analysis and source identification of heavy metals in the sediment of Poyang Lake in China. Sci. Total Environ. 2018, 621, 1433-1444.
Point 4: Lines 126–136: Was the accuracy of metal analysis verified using a proper standard reference material?
Response4: Thanks for reviewer’s question. The test accuracy was controlled by blank and parallel samples and controlled against the certified reference samples GSS-3(GBW07403) from the National Research Center (Beijing, China) to guarantee accuracy of the experimental measurements. Each sample was measured three times. The measurement accuracy was ±10%, and the measurement error was <5% between the measured and the verified values. We have supplemented this part in the article to address your concerns and hope that it is now clearer (lines158-163).
Point 5: Table 1 and lines 202–208: The significant figures for given values are too many. They will be at most three based on analytical errors of ICP-MS and ICP-OES. Moreover, correct the background values of Cd and Ba.
Response5: We guess that the reviewer may not understand the meaning of the data in lines 202-208. A total of 57 sediment samples were collected in this study, and these data (lines 202-208, now is lines233-239 (Revison)) are the range of the results of the tested element content. For example, in 57 samples, the content range of Cr element is 82.7-440mg/kg, the minimum is 82.7mg/kg, the maximum is 440mg/kg. In addition, we are very sorry that we made a mistake in entering the background values of Cd, Ba, Tl, the correct ones should be Cd (1.37), Ba (411), Tl (0.588) (mg/kg). Now we have modified them in Table 1.
Point 6: Do the authors mean that Ba is saturated with a sulfate or carbonate in the lake water? The authors should give evidence to support this.
Response6: Thanks for reviewer’s comment. Under the influence of sedimentary environment (such as water depth, redox, biological action, etc.), Ba ions entering the lake often interact with sulfate ions and carbonate ions, and then precipitate to form enrichment (Dehairs et al.,1980; Dymond et al.,1992; Pirrung et al.,2008). We have revised the text (lines 258-263).
References:
- Dehairs F,; Chesselet R,; Jedwab J. Discrete suspended particles of barite and the barium cycle in the open ocean. Earth and Planetary Science Letters.1980, 49(2): 528-550.
- Dymond, J.; Suess, E.; Lyle, M. Barium in deep‐sea sediment: A geochemical proxy for paleoproductivity. Paleoceanography. 1992, 7(2), 163-181.
- Pirrung M.; Illner P.; Matthießen J. Biogenic barium in surface sediments of the European Nordic Seas. Marine Geology. 2008, 250(1-2): 89-103.
Point7: Lines 233–234: I am surprised that the unit of element concentrations in the lake sediments is μg/kg (= ppb). Is this truly correct? If so, the element concentrations presented in Table 1 are abnormally low compared with the background values (unit: mg/kg = ppm). However, this will be impossible. Thus, I have doubts about the accuracy of analytical results. Was the accuracy of metal analysis verified using a proper standard reference material?
Response7: We are deeply thankful to the reviewer for raising this important point that the unit of element concentrations we presented was wrong. After repeated verifications and inspections, we found that we entered wrong units in the preliminary analysis, which led to some errors in the analysis results in the article. The correct unit should be ppm(mg/kg), and the correct conclusion is that except for Cd which is lower than the local background value, the content of other elements is much higher than the local background value. We are sorry for our mistakes. We have revised the text to address your concerns and hope that it is now clearer. Please see the revised manuscript. (lines 32-33, lines 267-278, lines 432-435)
Point8: Lines 275–277: I disagree with that As mainly derives from anthropogenic sources. The lake sediments are not polluted by As, because As concentration in the lake sediments is extremely lower than its background value.
Response8: Again, we apologized for our carelessness about the mistakes in the in the unit entry. In fact, the background value of As in the local area was 14 mg/kg, while the average As concentrations of the six lakes in the study area were 178.7, 228.7, 200.7, 219.5, 214.56, and 230.6 mg/kg (Table 1), which were much higher than the background value.
Point9: Lines 277–278: I do not understand that in nature, Ba mainly exists as either sulfuric or carbonic acid. In addition, what is the form of adsorption or mechanical mixing? Polite explanation is needed.
Response9: The trace element Ba mainly occurs in the terrigenous clastic and biogenic facies, and is obviously affected by the water depth of the sedimentary environment and the input of terrigenous materials. The main minerals of barium (Ba) in nature are barite (BaSO4) and witherite (BaCO3), and Ba is a typical lithophile element, which often occurs in potassium feldspar by isomorphic replacement with K (Dehairs et al.,1980; Dymond et al.,1992; Pirrung et al.,2008). In addition, we apologize for the inaccuracy of this statement (“the form of adsorption or mechanical mixing”). In fact, what this sentence wants to express is that Ba is different from other elements in the form of occurrence. The form of elements in sediments including exchangeable (adsorption state), carbonate binding, iron manganese oxide and organic matter bound (Tessier et al.,1979; Quevauviller et al.,1997; Quevauviller et al.,1998). (lines 314-315)
References:
- Dehairs F.; Chesselet R.; Jedwab J. Discrete suspended particles of barite and the barium cycle in the open ocean. Earth and Planetary Science Letters.1980, 49(2): 528-550.
- Dymond, J.; Suess, E.; Lyle, M. Barium in deep‐sea sediment: A geochemical proxy for paleoproductivity. Paleoceanography. 1992, 7(2), 163-181.
- Pirrung M.; Illner P.; Matthießen J. Biogenic barium in surface sediments of the European Nordic Seas. Marine Geology. 2008, 250(1-2): 89-103.
- Tessier A.; Campbell P G C.; Bisson M. Sequential extraction procedure for the speciation of particulate trace metals. Analytical Chemistry. 1979, 51(7): 844-851.
- Quevauviller P. Operationally defined extraction procedures for soil and sediment analysis standardization. Trends in Analytical Chemistry. 1998, 17(5): 289-298.
- Quevauviller P.; Lopezsanchez J F.; Rubio R, et al. Certification of trace metal extractable contents in a sediment reference material (CRM 601) following a three- step sequential extraction procedure. Science of the Total Environment. 1997, 205(2/3): 223-234.
Point10: The authors should give more evidence to support this source apportionment. What is the reason why the contribution of traffic emission to Cd is relatively large? Also, what is the reason why the contribution of atmospheric deposition and anthropogenic inputs to Ba is relatively large?
Response10: Thanks for the comment. We have added explanation part (lines 388-389, 394-396, 401-403). The study area was adjacent to the Qinghai-Tibet Railway and multiple national and provincial highways. And studies have shown that Cd is mostly derived from engine oils, abrasion of brake linings, and tearing of tires (Winther and Slentø, 2010; Guo et al., 2018). Therefore, we identified Cd in factor 2 as a source of traffic emission. Particles containing trace elements diffuse in air and are deposited, or transferred into sediment by dust deposition, precipitation, and runoff (Werkenthin et al., 2014; Wu et al., 2016). The study area was located in a transitional zone between aridity and semi-arid conditions, dust activity is intense. A small amount of volatile compounds (e.g., Pb, Cr, Zn, and other trace elements) are closely related to particles. During long-distance transportation, they are mainly deposited along with other particles (Wang et al., 2010). And studies have shown Zn, Pb, Cr, Ni, As, Ba, V, and Pb is known to be associated with traffic (Sternbeck et al., 2002; Lough et al., 2005; Hjortenkrans et al., 2006). Meanwhile, Ba is often used in diesel engines and other internal combustion engine cleaning agents (Wang et al., 2017). Therefore, we assume that atmospheric deposition and anthropogenic input contribute greatly to Ba.
References:
- Winther, M.; Slentø, E. Heavy Metal Emissions for Danish Road Transport. Aarhus University, National Environmental Research Institute. 2010.
- Guo, B.; Liu, Y.; Zhang, F.; Hou, J.; Zhang, H.; Li, C. Heavy metals in the surface sediments of lakes on the Tibetan Plateau, China. Environ. Sci. Pollut. Res. 2018, 25(4), 3695-3707.
- Wang, X.; Yang, H.; Gong, P.; Zhao, X.; Wu, G.; Turner, S.; Yao, T. One century sedimentary records of polycyclic aromatic hydrocarbons, mercury and trace elements in the Qinghai Lake, Tibetan Plateau. Environ. Pollut. 2010, 158(10), 3065-3070.
- Werkenthin, M.; Kluge, B.; Wessolek, G. Metals in European roadside soils and soil solution–a review. Environ. Pollut. 2014,189, 98–110.
- Wu, J.; Duan, D.; Lu, J.; Luo, Y.; Wen, X.; Guo, X.; Boman, B. J. Inorganic pollution around the Qinghai-Tibet Plateau: An overview of the current observations. Sci. Total Environ. 2016, 550, 628-636.
- Sternbeck, J.; Sjödin, Å.; Andréasson, K. Metal emissions from road traffic and the influence of resuspension—results from two tunnel studies. Atmos. Environ. 2002,36 (30), 4735–4744.
- Lough, G.C.; Schauer, J.J.; Park, J.-S.; Shafer, M.M.; DeMinter, J.T.;Weinstein, J.P. Emissions ofmetals associatedwithmotor vehicle roadways. Environ. Sci. Technol. 2005, 39 (3), 826–836.
- Hjortenkrans, D.; Bergbäck, B.; Häggerud, A. New metal emission patterns in road traffic environments. Environ. Monit. Assess. 2006, 117 (1–3), 85–98.
- Wang, G.; Zeng, C.; Zhang, F.; Zhang, Y.; Scott, C. A.; Yan, X. Traffic-related trace elements in soils along six highway segments on the Tibetan Plateau: Influence factors and spatial variation. Science of the Total Environment.2017, 581: 811-821.
We would like to thank the referee again for taking the time to review our manuscript.

Reviewer 2 Report
A brief summary
A review of the manuscript entitled: „Geochemical and Statistical Analyses of Trace Elements in Lake Sediments from Qaidam Basin, Qinghai-Tibet Plateau: Distribution Characteristics and Source Apportionment”is interesting and promising.
Based on this general evaluation and the specific comments, reported below, I recommend a minor revisions of the manuscript. I have few comments and suggestions, which might improve the manuscript.
Specific comment
Material and methods
The method of data collection needs to be described in greater detail. From which zone of the lake sediment samples were taken? Shallow zone or deep zone? Based one the supplementary material – sediments were collected from different zones of the lakes. What layer of sediment was taken?
Statistical analysis
Authors should describe the coefficient of variations (CVs).
Results
Table 1 – ‘the trace element concentrations (μg/kg) in sediments (mg/kg) and comparison with other selected water systems around the world (mg/kg)’ expressed in different units. The different units can make it difficult for readers to compare.
Author Response
Thanks for your valuable and helpful comments. We have responded comments carefully and made the necessary correction point by point. All of the revised part in our revised manuscript has been highlighted in red.
Response to Reviewer 2 Comments
Point 1: A review of the manuscript entitled: “Geochemical and Statistical Analyses of Trace Elements in Lake Sediments from Qaidam Basin, Qinghai-Tibet Plateau: Distribution Characteristics and Source Apportionment” is interesting and promising.
Based on this general evaluation and the specific comments, reported below, I recommend a minor revisions of the manuscript. I have few comments and suggestions, which might improve the manuscript.
Response 1: We are grateful to the reviewer by this great suggestion. We have carefully considered the suggestion of Reviewer and tried our best to improve and made some changes in the manuscript.
Point 2: Material and methods
The method of data collection needs to be described in greater detail. From which zone of the lake sediment samples were taken? Shallow zone or deep zone? Based one the supplementary material – sediments were collected from different zones of the lakes. What layer of sediment was taken?
Response 2: Thanks for your comment. We have described and supplemented the sampling process in detail, including water depth, sampling area, etc. And hope that it is now clearer. Please see the revised manuscript. (lines 117-110) In addition, sediment with a thickness of 4-10 cm on the surface layer was taken in study area.
Point 3: Statistical analysis
Authors should describe the coefficient of variations (CVs).
Response 3: Thanks for the reviewer’s kind advice. The coefficient of variation (CVs) can be used to compare the degree of dispersion of the data. It is the ratio of the standard deviation of the original data to the mean of the original data. We have added the part to address your concerns and hope that it is now clearer. Please see lines 226-228.
Point 4: Results
Table 1 – ‘the trace element concentrations (μg/kg) in sediments (mg/kg) and comparison with other selected water systems around the world (mg/kg)’ expressed in different units. The different units can make it difficult for readers to compare.
Response4: Thanks for the reviewer’s kind advice. We have unified the unit of content data in Table 1 to mg/kg, so that readers can make a clear and intuitive comparison.
We would like to thank the referee again for taking the time to review our manuscript.

Reviewer 3 Report
I reviewed the manuscript #1558804 submitted to International journal of Environmental Research and Public Health
with the title "Geochemical and statistical analyses of trace elements in lake sediments from Qaidam Basin, Qinghai-Tibet Plateau: Distribution characteristics and source apportionment"
by He, Wei, Huan, Wang, Qin, Li, Shan, Fan and Du
This manuscript reports amounts of selected elements in sediments from six lakes in the Qaida Basin, China. The authors compare elements from each lake and provide hypotheses regarding the source of certain elements. The manuscript well organized and written. The many figures illustrate different components of the analysis. Although analysis and assessment of lake sediments is not my primary area of expertise, the material presented is easily understandable and generally is an adequate description of the research project. However, I found the description of sampling in methods lacking certain desirable information. Although sediment sample processing is described in detail, no information is provided on how the samples were obtained in the paragraph between lines 110 and 115.
For section 2.2, first paragraph, please provide additional information on:
-were sample sites randomly or subjectively selected?
-describe the grab sample equipment, such as a core or other?
-how deep in sediments were samples collected?
-where in lakes were samples taken? near shore line or x distance from shore?
-were samples in each lake collected at one site, or at multiple sites?
-were samples collected by one person or various people?
-were all lakes sampled during one month or over many months?
Author Response
Thanks for your valuable and helpful comments. We have responded comments carefully and made the necessary correction point by point. All of the revised part in our revised manuscript has been highlighted in red.
Please kindly see the attachment.

Reviewer 4 Report
He et al. evaluated 57 sediment samples for (V, Cr, Ni, Cu, Zn, As, Ba, Tl, Cd, Pb, and U and the results showed that Ba, Zn, V, and Cr had a higher content and a wider distribution relative to the other tested elements with a strong correlation revealing the source of similarity that being atmospheric deposition and anthropogenic inputs.
Line 48-50 More data regarding sediments as a classic indicator of pollution should be added.
I couldn't see any data regarding zoobenthos as a biomarker regarding these elements, this should be also added.
Materials and methods
GIS coordinates of the sampling sites should be added
Line 131-133 State the protocol used in a step by step manner
Author Response
Thanks for your valuable and helpful comments. We have responded comments carefully and made the necessary correction point by point. All of the revised part in our revised manuscript has been highlighted in red.
Response to Reviewer 4 Comments
Point 1: He et al. evaluated 57 sediment samples for (V, Cr, Ni, Cu, Zn, As, Ba, Tl, Cd, Pb, and U and the results showed that Ba, Zn, V, and Cr had a higher content and a wider distribution relative to the other tested elements with a strong correlation revealing the source of similarity that being atmospheric deposition and anthropogenic inputs.
Line 48-50 More data regarding sediments as a classic indicator of pollution should be added.
Response 1: Thanks for your kind advice. There is indeed a lack of sufficient evidence to illustrate this view, we have revised the text and added the relevant literature. (lines54-56)
References:
- Guo, B.; Liu, Y.; Zhang, F.; Hou, J.; Zhang, H.; Li, C. Heavy metals in the surface sediments of lakes on the Tibetan Plateau, China. Environ. Sci. Pollut. Res. 2018, 25(4), 3695-3707.
- Fatoki O S.; Mathabatha S. An assessment of heavy metal pollution in the East London and Port Elizabeth harbours. Water Sa 2001, 27(2): 233-240.
- Vandecasteele B.; Quataert P.; De Vos B.; Tack, F. M. Assessment of the pollution status of alluvial plains: a case study for the dredged sediment-derived soils along the Leie river. Archives of environmental contamination and toxicology.2004, 47(1): 14-22.
- Zan F.; Huo S.; Xi B.; Su, J.; Li, X., Zhang, J.; Yeager, K. M. A 100 year sedimentary record of heavy metal pollution in a shallow eutrophic lake, Lake Chaohu, China. Journal of Environmental Monitoring, 2011, 13(10): 2788-2797.
- Yuan G L.; Liu C.; Chen L.; Yang, Z. Inputting history of heavy metals into the inland lake recorded in sediment profiles: Po-yang Lake in China. Journal of hazardous materials. 2011, 185(1): 336-345.
Point 2: I couldn't see any data regarding zoobenthos as a biomarker regarding these elements, this should be also added.
Response 2: We are grateful to the reviewer by this great suggestion. We explain for this: This study attempts to use a geochemical and statistical approach to clarify the degree and distribution of trace element pollution in the sediments of the six identified lakes in the Qinghai-Tibet Plateau and determine the correlation between different trace elements and deduce the artificial sources and/or natural sources of any identified trace elements in sampled lake sediments. Benthic indicators can sensitively and quickly characterize sediment toxicity, we have not studied them in detail. But this is a great inspiration for us, and we will add biomarker in our future research work.
Point 3: Materials and methods
GIS coordinates of the sampling sites should be added
Response 3: Thanks for the reviewer’s kind advice. GIS coordinates of the sampling sites have been added. Please see line 121,124.125,128, 131,137.
Point 4: Line 131-133 State the protocol used in a step by step manner
Response4: Thank you for your careful suggestions. We have revised the text to address your concerns and hope that it is now clearer. (lines 156-163)
We would like to thank the referee again for taking the time to review our manuscript.

Round 2
Reviewer 1 Report
I think that the revised manuscript has been satisfactorily improved. Hence, this is acceptable for publication; however, the authors should reconsider on the following points.
Table 1 and Lines 230-236: The number of significant figures for given values will be at most three based on analytical errors of ICP-OES and ICP-MS.
Line 310: Correct as follows
"As mentioned earlier, Ba naturally exists as either a sulfate or carbonate, different from other elements in the form of the occurrence."
Author Response
Point 1: Table 1 and Lines 230-236: The number of significant figures for given values will be at most three based on analytical errors of ICP-OES and ICP-MS.
Response1: We have revised theTable 1 and Lines 230-236 to address your concerns and hope that it is now clearer. We would like to thank you again for your time in reviewing this manuscript.
Point 2: Line 310: Correct as follows
"As mentioned earlier, Ba naturally exists as either a sulfate or carbonate, different from other elements in the form of the occurrence."
Response2: Thank you for your suggestion, it is indeed much clearer than our previous statement.

Reviewer 4 Report
The authors have revised their work accordingly
Author Response
We would like to thank you again for your time in reviewing this manuscript.