# Supplementary material for: Geochemical and Statistical Analyses of Trace Elements in Lake Sediments from Qaidam Basin, Qinghai-Tibet Plateau: Distribution Characteristics and Source Apportionment
Source: Int J Environ Res Public Health. 2022 Feb 18;19(4):2341. doi: 10.3390/ijerph19042341 (PMC8872242; doi:10.3390/ijerph19042341)
Supplement: Supplementary file 1 [file ijerph-19-02341-s001.zip › ijerph-1558804-supplementary.pdf]

International Journal of
Environmental Research and Public Health

MDPI

Supplementary Material- Geochemical and Statistical Analysis of Trace Elements in Lake Sediments in Qaidam Basin, Qinghai-Tibet Plateau: Revelation for Distribution Characters and Source Apportionment

Haifang He [1,3,4], Haicheng Wei [1,3,*], Yong Wang [2], Lingqing Wang [2,*], Zhanjie Qin [1,3], Qingkuan Li [1,3], Fashou Shan[1,3], Qishun Fan[1,3] and Yongsheng Du [1,3]

[1] Key Laboratory of Comprehensive and Highly Efficient Utilization of Salt Lake Resources, Qinghai Institute of Salt Lakes, Chinese Academy of Sciences, Xining 810008, China; hehaifang19@mails.ucas.ac.cn (H.H.); hcwei@isl.ac.cn (H.W); qinzhanjie@isl.ac.cn (Z.Q.); liqingkuan@isl.ac.cn (Q.L.); shanfsh@isl.ac.cn (F.S.); qsfan@isl.ac.cn(Q.F.); dys@isl.ac.cn (Y.D.)

[2] Institute of Geographical Sciences and Natural Resources Research, Chinese Academy of Sciences, Beijing, ,100101, China; wangy@igsnrr.ac.cn (Y.W.); wanglq@igsnrr.ac.cn (L.W.)

[3] Qinghai Provincial Key Laboratory of Geology and Environment of Salt Lakes, Xining 810008, China; hehaifang19@mails.ucas.ac.cn (H.H.); hcwei@isl.ac.cn (H.W); qinzhanjie@isl.ac.cn (Z.Q.); liqingkuan@isl.ac.cn (Q.L.); shanfsh@isl.ac.cn (F.S.); qsfan@isl.ac.cn(Q.F.); dys@isl.ac.cn (Y.D.)

[4] University of Chinese Academy of Sciences, Beijing 100049, China; hehaifang19@mails.ucas.ac.cn (H.H.)

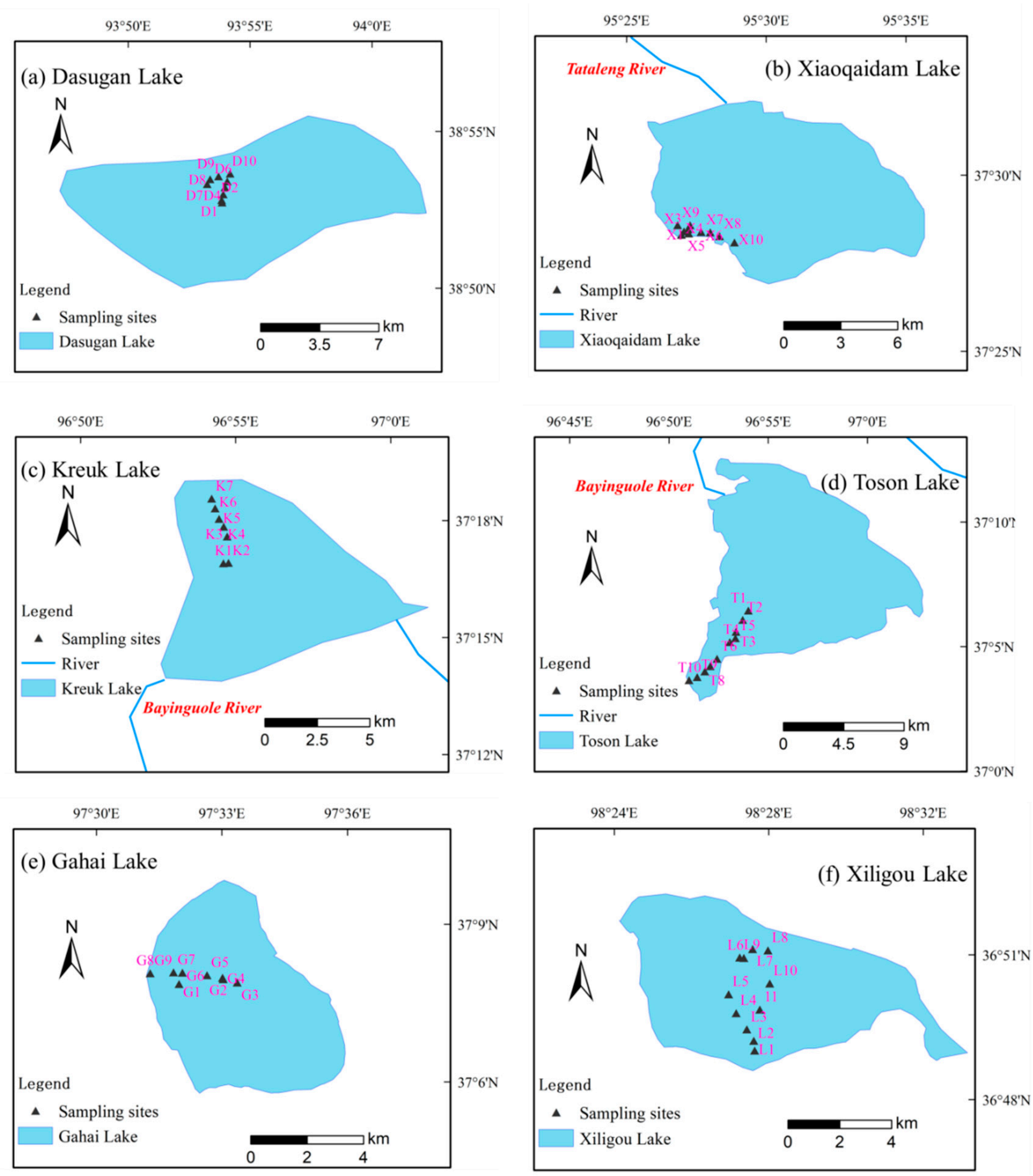

Figure S1. Sampling points of the six lakes in Qaidam Basin, QingHai-Tibet Platea.
